# Comparing outcomes between culture-positive and culture-negative septic shock in a PICU: A retrospective cohort study

**DOI:** 10.3389/fped.2022.1001565

**Published:** 2022-10-13

**Authors:** Haixin Huang, Jian Chen, Hongxing Dang, Chengjun Liu, Yue-qiang Fu

**Affiliations:** Department of Critical Care Medicine, Children’s Hospital of Chongqing Medical University, National Clinical Research Center for Child Health and Disorders, Ministry of Education Key Laboratory of Child Development and Disorders, Chongqing Key Laboratory of Pediatrics, Chongqing, China

**Keywords:** septic shock, children, culture negative, culture positive, mortality

## Abstract

**Background:**

We assessed the outcomes and characteristics of culture-negative septic shock (CNSS) and culture-positive septic shock (CPSS) in pediatric intensive care unit (PICU).

**Methods:**

We performed a retrospective study on the data of children admitted to the PICU due to septic shock between January 2018 and December 2021. The primary outcome was in-hospital mortality. The secondary outcomes were the length of stay (LOS) of hospital, the need for mechanical ventilation (MV) and continue renal replacement therapy (CRRT).

**Results:**

Overall, 238 patients were enrolled. 114 patients (47.9%) had positive cultures (60 blood samples, 41 sputum samples, 17 pus samples, and 19 others), 18 of whom were cultured positive at two sites, 1 at three sites, and 3 had two different types of bacteria at same site. The in-hospital mortality was 47.1%. There were no significant differences in the in-hospital mortality (47.6% vs. 46.5%, *P* = 0.866), PRISM-III score (10 vs. 12, *P* = 0.409), PIM-3 score (0.08 vs. 0.07, *P* = 0.845), pSOFA score (10 vs. 10, *P* = 0.677) or the need for MV (64.5% vs. 68.4%, *P* = 0.524) and CRRT (29.8% vs. 34.2%, *P *= 0.470) between the CNSS group and the CPSS group. The Procalcitonin (8.89 ng/ml vs. 28.39 ng/ml, *P* = 0.001) and C-reactive protein (28 mg/L vs. 58 mg/L, *P* = 0.001) levels were significantly lower in the CNSS group than in the CPSS group, while WBC count (9.03 × 10^9^/L vs. 5.02 × 10^9^/L, *P* = 0.002) and serum sodium (137 mmol/L vs. 132 mmol/L, *P* = 0.001) was significantly higher in CNSS. The LOS of hospital was significantly longer (16 days vs. 11 days, *P* = 0.011) in the CPSS group than in the CNSS group, while the LOS of PICU (5 days vs. 4 days, *P* = 0.094) stay was not significantly different.

**Conclusion:**

Compared with children with CNSS, children with CPSS had higher PCT and CRP levels, but lower WBC count. Children with CPSS had longer LOS of hospital. However, positive or negative culture results were not associated with in-hospital mortality, the LOS of PICU, the need for MV or CRRT in children with septic shock.

## Introduction

Sepsis is a leading cause of health loss worldwide and consumes significant medical resources (1). Children are the main population of patients with sepsis, with 1.2 million cases each year (2). Sepsis is diagnosed in 4%–8% of patients in the PICU, and the mortality rate in children with sepsis ranges from 4% to 50% (3), but it is influenced by factors such as disease severity, risk factors, geographic location and available resources. Taking into account the influence of resources and other factors, a guideline (4) published this year on sepsis in children mentioned that the mortality rate from sepsis in low- and middle-income countries was higher than in high-income countries (31.7% vs. 19.3%). Regions located in low- and middle-income countries had a greater risk of death than high-income regions (Africa 7.89, Asia 3.81, South America 2.91). Septic shock is a serious complication of sepsis. In addition to antibiotics, organ function replacement therapy, such as mechanical ventilation and/or renal replacement therapy, is usually required in septic children who suffer from refractory shock and/or multiple organ dysfunction syndrome.

When treating sepsis, the current guidelines recommend obtaining cultures before initiating antimicrobial therapy (3). Physicians strictly follow these guidelines and collect biological specimens for culture to identify pathogens. However, in many cases, treatment of sepsis is initiated on the basis of clinical suspicion, pending the outcome of ongoing evaluations and laboratory results. Once the pathogen(s) and sensitivities are available according to the culture, targeted antimicrobial therapy can be achieved, which may reduce the blindness of treatment. Unfortunately, the identification of specific microorganisms by culture remains challenging (5) because of the unsatisfactory positive rate of culture in the clinic. Studies have found that the proportion of culture-negative cases in adult sepsis or septic shock patients ranges from 28% to 69.4% (6, 7).

Although the culture of biological specimens is an indispensable part of the initial therapy of sepsis, the impact of culture results on mortality, therapy requirements and other clinical outcomes in pediatrics is still not well studied. Exploratory studies were made in the adult field. Kim et al. (8) showed that neither culture-negativity nor culture-positivity was associated with mechanical ventilation duration or mortality in adult patients with septic shock. One meta-analysis also reported that culture results were not associated with mortality in adult patients with sepsis or septic shock, while the hospital length of stay and mechanical ventilation duration were both longer in culture-positive septic patients (9). Hazwani et al. (10) focused on the role of blood culture and reported that the mortality of culture-positive septic children was significantly higher than that of culture-negative septic children, while their research only focused on blood culture and did not take into account the results of other biological specimens culture. Up to now, the relationship between the culture results of biological specimens and clinical outcome is rarely explored in children with septic shock.

We hypothesized that children with positive cultures would have worse clinical outcomes than children with negative cultures, as positive cultures may represent higher pathogen load and hint at more severe illness scores for patients with septic shock. To verify this, we compared the clinical parameters, therapy requirements and outcomes of culture-negative septic shock (CNSS) and culture-positive septic shock (CPSS) children.

## Methods

### Study design

We performed a retrospective cohort study to analyze the clinical data and outcomes of children with septic shock admitted to the PICU of Children's Hospital of Chongqing Medical University from January 1, 2018, to December 31, 2021. The PICU is a comprehensive central intensive care unit with 80 open beds divided into a medical subunit and a surgical subunit. The Institutional Review Board of Children's Hospital of Chongqing Medical University approved this study. The data of patients were anonymized and deidentified prior to analysis. The requirement for informed consent was waived due to the retrospective nature of this study.

Infection was clinically diagnosed by the children's attending physicians according to the clinical manifestation, laboratory examination and imaging. Septic shock was diagnosed as severe infection leading to cardiovascular dysfunction (including hypotension, need for treatment with vasoactive medication, or impaired perfusion), referring to the 2017 clinical practice and 2020 guidelines for children (3, 11). The septic shock patients were given the following procedure to identify pathogens in our hospital. Duplicate blood cultures were drawn from two sites on admission, and the volume of blood collected was determined by the patient's weight, with cutoffs of 1, 2, 12.7, and 36.3 kg (12). And the samples of other biological specimens according to the suspicious site of infection were also collected for culture, including sputum, pleural effusion, ascites, urine, feces, pus and others.

Patients were identified according to the discharge diagnosis from the hospital electronic medical databases and admission to the PICU due to septic shock. We excluded the patients diagnosed with septic shock in other departments of the hospital but not transferred to the PICU and the patients with combined viral infections. We also excluded the patients from surgery department undergoing abdominal surgery transferred to PICU for postoperative anesthetic ventilation support only and returned to surgery department in the next day.

### Data collection and definition of variables

The culture results were collected. The following demographic and clinical data were examined: age, sex, comorbidity, white blood cell (WBC), hemoglobin (Hb), platelet (PLT), Procalcitonin (PCT), C-reactive protein (CRP), lactate, glucose, bilirubin, albumin, creatinine, urea, alanine aminotransferase (ALT), international normalized ratio (INR), serum sodium (Na^+^) and serum potassium (K^+^). The blood samples was collected at admission, such as PCT, CRP and other blood parameters.

Severity of illness scores, such as pediatric index of mortality (PIM)-3 (13), pediatric risk of mortality (PRISM)-III (14), and pediatric sequential organ failure assessment (pSOFA) (15) scores, were obtained. The need for continued renal replacement therapy (CRRT) and mechanical ventilation (MV) was collected, and the duration of CRRT and MV was also calculated.

### Outcomes

The primary outcome was in-hospital mortality at any time. The secondary outcomes were the length of PICU stay, the length of hospital stay, and the need for MV and CRRT. To account for death as a competing outcome, PICU-free days and hospital-free days with a maximum of 14 and 28 days were used, respectively.

### Statistical analysis

Statistical analysis was performed with SPSS Statistics for Windows, Version 26 (IBM Corp., Armonk, NY, USA). Data were stratified by culture results (i.e., culture-negative and -positive). Continuous variables are expressed as the medians (interquartile range), and categorical variables are shown as the counts (frequency or percentage). Continuous variables were compared using the Mann-Whitney U test, and categorical variables were analyzed using the chi-squared or Fisher's exact test. Kaplan-Meier survival curves were subjected to the log-rank test based on culture results. *P* values < 0.05 were considered to be statistically significant.

## Results

In total, 238 children with septic shock were enrolled during the study period, which included 124 (52.1%) in the CNSS group and 114 (47.9%) in the CPSS group. The clinical characteristics of the children according to the culture results are listed in Table 1. Children in the CNSS group were younger than those in the CPSS group (36 vs. 48 months), but the difference was not statistically significant (*P* = 0.700). In terms of laboratory tests, there was no significant difference in bilirubin (6.65 vs. 9.20 μmol/L), albumin (26.9 vs. 24.9 g/L), lactate (2.0 vs. 2.0 mmol/L), creatinine (46.0 vs. 48.0 μmol/L), glucose (6.0 vs. 6.2 mmol/L), urea (5.91 vs. 6.60 mmol/L), INR (1.37 vs. 1.44), or K^+^ (3.6 vs. 3.5 mmol/L) between the CNSS group and the CPSS group. There were also no significant differences in the PIM-3, PRISM-III and pSOFA scores. Moreover, the requirements of CRRT and MV were similar between the two groups. Although the days of CRRT and MV in CPSS group were greater than those in CNSS group, the difference was not statistically significant.

**Table 1:** Demographics, clinical characteristics of children with septic shock according to the culture results.

Characteristics	Total (*N* = 238)	CNSS (*n* = 124)	CPSS (*n* = 114)	*P*
Age (month), M (IQR)	36 (9∼132)	36 (9∼132)	48 (9.5∼126)	0.700
Gender (male), *n* (%)	127 (53.4%)	67 (54.0%)	60 (52.6%)	0.829
Comorbidity, *n* (%)				
Hematological malignancy	45 (18.9%)	17 (13.7%)	28 (24.6%)	0.033
Tumor	11 (4.6%	6 (4.8%)	5 (4.4%)	0.868
Immunodeficiency	12 (5.0%)	5 (4.0%)	7 (6.1%)	0.458
Rheumatism	24 (10.1%)	16 (12.9)	8 (7.0%)	0.132
PRISM-III, M (IQR)	11 (6∼17)	10 (5∼18)	12 (7∼17)	0.409
PIM-3, M (IQR)	0.08 (0.05∼0.15)	0.08 (0.04∼0.17)	0.07 (0.05∼0.13)	0.845
pSOFA, M (IQR)	10 (6∼14)	10 (6∼14)	10 (6∼14)	0.677
WBC (x10^9^/l), M (IQR)	6.76 (2.34∼14.68)	9.03 (3.79∼16.38)	5.02 (0.89∼11.91)	0.002
Hb (g/l), M (IQR)	95 (77∼114)	98 (82∼119)	88 (72∼106)	0.002
PLT (x10^9^/l), M (IQR)	111 (33∼258)	127 (46∼302)	85 (19∼187)	0.001
CRP (mg/l), M (IQR)	44 (15∼76)	28 (4∼65)	58 (23∼79)	0.001
PCT (ng/ml), M (IQR)	17.42 (2.85∼70.93)	8.89 (2.00∼64.14)	28.39 (7.80∼71.65)	0.001
INR, M (IQR)	1.40 (1.17∼1.83)	1.37 (1.14∼1.90)	1.44 (1.21∼1.82)	0.365
Lactate (mmol/L), M (IQR)	2.0 (1.0∼4.4)	2.0 (1.0∼4.5)	2.0 (1.0∼4.4)	0.856
Glucose (mmol/L), M (IQR)	6.1 (4.8∼7.9)	6.0 (4.7∼8.2)	6.2 (4.9∼7.7)	0.970
K^+^ (mmol/L), M (IQR)	3.6 (3.2∼4.2)	3.6 (3.2∼4.4)	3.5 (3.1∼4.1)	0.168
Na^+^ (mmol/L), M (IQR)	135 (130-139)	137 (132-140)	132 (129-138)	0.001
Bilirubin (*μ*mol/L), M (IQR)	7.90 (3.70∼18.15)	6.65 (3.50∼15.48)	9.20 (4.05∼22.60)	0.053
ALT (U/L), M (IQR)	46.1 (28.5∼103.5)	46.5 (30.2∼111.5)	44.5 (25.2∼93.8)	0.187
Albumin (g/L), M (IQR)	25.7 (23.0∼31.9)	26.9 (23.3∼32.4)	24.9 (22.4∼30.8)	0.053
Creatinine (μmoI/L), M (IQR)	47.0 (27.5∼83.2)	46.0 (27.6∼97.8)	48.0 (26.9∼79.4)	0.804
Urea (mmol/L), M (IQR)	6.34 (3.97∼10.19)	5.91 (3.92∼10.72)	6.60 (4.18∼9.91)	0.621
Antibiotic use before PICU, *n* (%)	158 (66.4%)	81 (65.3%)	77 (67.5%)	0.717
Use of vasoactive drugs within 24 h, *n* (%)	183 (76.9%)	93 (75.0%)	90 (78.9%)	0.470
Need of CRRT, *n* (%)	76 (31.9%)	37 (29.8%)	39 (34.2%)	0.470
The length of CRRT (days), M (IQR)	3 (1∼4)	2 (1∼4)	3 (2∼4)	0.348
Need of MV, *n* (%)	158 (66.4%)	80 (64.5%)	78 (68.4%)	0.524
The length of MV (days), M (IQR)	4 (1∼9)	4 (1∼9)	5 (2∼10)	0.201

CNSS, culture-negative septic shock; CPSS, culture-positive septic shock; CRP, C-reactive protein; CRRT, continuous renal replacement therapy; Hb, hemoglobin; INR, international normalized ratio; IQR, interquartile range; K^+^, blood potassium; M, median; MV, mechanical ventilation; Na+, serum sodium; PLT, platelet; PCT, procalcitonin; pSOFA, pediatric sequential organ failure assessment; WBC, white blood cells.

The PCT (8.89 vs. 28.39 ng/ml, *P *= 0.001) and CRP (28 vs. 58 mg/L, *P *= 0.001) levels were significantly lower in the CNSS group than in the CPSS group, while the reverse was true for the WBC (9.03 vs. 5.02  × 10^9^/L, *P *= 0.002), PLT (127 vs. 85  × 10^9^/L, *P *= 0.001), Hb (98 vs. 88 g/L, *P *= 0.002) counts and serum sodium (137 vs.132 mmol/L, *P *= 0.001) (Table 1).

To investigate whether these differences were due to the different compositions of patients with hematologic malignancy (13.7 vs. 24.6%, *P =* 0.033) in the two groups, patients with hematologic malignancy (*n* = 45) were excluded, and characteristic data were reanalyzed (Supplementary Table S1). Compared with the CNSS group, PCT (9.62 vs. 34.63 ng/ml, *P *= 0.003) and CRP (26 vs. 59 mg/L, *P *= 0.002) in the CPSS group were still significantly higher, while WBC (10.14 vs. 8.03  × 10^9^/L, *P *= 0.046) and serum sodium (137 vs.132 mmol/L, *P  *= 0.003) was still significantly lower in the CPSS group after exclusion (Supplementary Table S1). However, PLT (174 vs. 143  × 10^9^/L, *P *= 0.061) and Hb (100 vs. 96 g/L, *P *= 0.108) were not significantly different between the two groups (Supplementary Table S1).

As shown in Table 2, there was no significant difference (47.6 vs. 46.5%, *P *= 0.866) in in-hospital mortality between the CNSS group and the CPSS group. The length of stay (LOS) of hospital stay was significantly longer (16 vs. 11 days, *P *= 0.011) in the CPSS group than in the CNSS group, while the length of PICU stay was not significantly different (5 vs. 4 days, *P *= 0.094). The CPSS group had fewer hospital-free days (*P *= 0.025).

**Table 2:** Clinical outcomes according to the culture results.

Characteristics	Total (*N* = 238)	CNSS (*n* = 124)	CPSS (*n* = 114)	*P*
LOS of PICU (days), M (IQR)	5 (2∼10)	4 (1∼9)	5 (3∼10)	0.094
LOS of hospital (days), M (IQR)	14 (4∼27)	11 (2∼23)	16 (7∼31)	0.011
14-day PICU-free days, M (IQR)	0 (0∼8)	0 (0∼9)	0 (0∼9)	0.443
28-day hospital-free days, M (IQR)	0 (0∼7)	0 (0∼11)	0 (0∼3)	0.025
In-hospital mortality, n (%)	112 (47.1%)	59 (47.6%)	53 (46.5%)	0.866

IQR, interquartile range; LOS, length of stay; M, median; PICU, pediatric intensive care unit.

After excluding 45 patients with hematologic malignancy, there was still no significant difference (43.0% vs. 41.9%, *P *= 0.875) in in-hospital mortality between the two groups (Supplementary Table S2). The LOS of PICU and hospital was both significantly shorter (4 vs. 7 days, *P *= 0.004; 10 vs. 15 days, *P *= 0.007) in the CNSS group than in the CPSS group (Supplementary Table S2).

Organ dysfunction was similar between the two groups (Table 3). The proportion of respiratory failure was the highest (68.1%), followed by AKI (20.2%) and gastrointestinal bleeding (16.0%). The proportion of ARDS was more elevated in the CPSS group than in the CNSS group.

**Table 3:** Organ dysfunction of CNSS group and CPSS group.

Organ dysfunction	Total (*N* = 238)	CNSS (*n* = 124)	CPSS (*n* = 114)	*P*
Respiratory failure, *n* (%)	162 (68.1%)	81 (65.3%)	81 (71.1%)	0.344
AKI, *n* (%)	48 (20.2%)	25 (20.2%)	23 (20.2%)	0.998
Gastrointestinal bleeding, *n* (%)	38 (16.0%)	16 (12.9%)	22 (19.3%)	0.178
DIC, *n* (%)	21 (8.8%)	10 (8.1%)	11 (9.6%)	0.667

AKI, acute kidney injury; DIC, disseminated intravascular coagulation.

Infections of the respiratory system (37.0%) and digestive system (34.5%) were dominant in children with septic shock (Table 4). The proportion of digestive system infections was significantly higher in the CNSS group than in the CPSS group (42.7 vs. 25.4%, *P *= 0.005), while the reverse was true for skin and soft tissue infections (3.2 vs. 10.5%, *P *= 0.025, Table 4).

**Table 4:** Primary infection sites.

Infection site	Total (*n* = 238)	CNSS (*n* = 124)	CPSS (*n* = 114)	*P*
Respiratory system, *n* (%)	88 (37.0%)	41 (33.1%)	47 (41.2%)	0.192
Digestive system, *n* (%)	82 (34.5%)	53 (42.7%)	29 (25.4%)	0.005
Skin and soft tissue, *n* (%)	16 (6.7%)	4 (3.2%)	12 (10.5%)	0.025
Nervous system, *n* (%)	12 (5.0%)	7 (5.6%)	5 (4.4%)	0.657
Urinary system, *n* (%)	1 (0.4%)	1 (0.8%)	0 (0.0%)	-
Others, *n* (%)	21 (8.8%)	10 (8.1%)	11 (9.6%)	0.667
Unknown, *n* (%)	18 (7.6%)	8 (6.5%)	10 (8.8)	0.499

The pathogens of the positive cultures are shown in Table 5. The most frequent bacterium was *Staphylococcus* species (21.9%). However, gram-negative bacteria accounted for 51.8% of all bacteria, and *Klebsiella* species were the most common gram-negative bacteria. 56 children were infected with gram-negative bacteria only, and 38 children were infected with gram-positive bacteria only. There was no significant difference in PCT (28.4 vs. 28.9 ng/ml, *P* = 0.862) between children infected with gram-negative bacteria only and those infected with gram-positive bacteria only. However, children infected with gram-positive bacteria only had significantly elevated CRP (69.5 vs. 50.5 mg/L, *P* = 0.03), compared with children infected with gram-negative bacteria only.

**Table 5:** Frequency of bacteria for the positive cultures.

	Frequency (%)	Blood	Sputum	Pus	Urine	Etc^a^
Gram-positive, *n* (%)	50 (36.5%)					
* Staphylococcus* species	30 (21.9%)	15	11	2	-	2
* Streptococcus* species	16 (11.7%)	7	3	4	-	2
* *Others	4 (2.9%)	-	-	1	1	2
Gram-negative, *n* (%)	71 (51.8%)					
* Klebsiella* species	18 (13.1%)	12	2	2	-	2
* Pseudomonas aeruginosa*	15 (10.9%)	7	5	2	-	1
* Escherichia coli*	14 (10.2%)	6	2	4	1	1
* Acinetobacter baumannii*	10 (7.3%)	1	8	-	-	1
* *Others	14 (10.2%)	7	5	-	-	2
Fungus, *n* (%)	16 (11.7%)					
* Candida*	15 (10.9%)	5	5	2	-	3
* Talaromyces marneffei*	1 (0.7%)	-	-	-	-	1

Others included: Ralstonia mannitollytica, Burkholderia cepacia, Aeromonas hydrophila, Neisseria meningitides, Haemophilus influenzae, Enterobacter cloacae, Stenotrophomonas maltophilia, fecal Enterococcus.

^a^ Etc included: Pleural effusion, Ascites, Cerebrospinal fluid.

**Figure 1:**
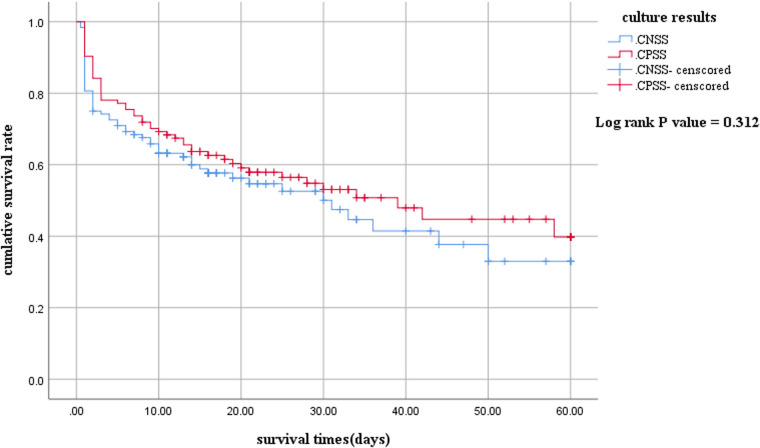
Kaplan-Meier survival curves between children with culture-negative and culture-positive septic shock. The survival curves censored to 60 days. CNSS, culture-negative septic shock; CPSS, culture-positive septic shock.

The 60-day survival rate in the CNSS group was slightly lower than that in the CPSS group (Figure 1). However, the difference was not statistically significant (log-rank *P *= 0.312).

## Discussion

In the diagnosis and treatment of septic shock in children, it is routine to identify pathogenic microorganisms by culture. However, the positive rate of culture is limited, and the impact of positive or negative culture results on the treatment and prognosis of children with septic shock remains unclear. Our study showed that: (1) approximately 52.1% of septic shock children had negative culture results; (2) the in-hospital mortality of CNSS patients and CPSS patients was very similar, while the length of hospital stay of CPSS patients was longer than that of CNSS patients; and (3) PCT, CRP and Na^+^ in the two groups were significantly different.

This study showed fairly common negative culture results in children with septic shock, which was consistent with the previous findings of several retrospective studies of adult patients with septic shock or severe sepsis (7, 9, 16, 17). In our study, there were several reasons for the negative culture results. First, the positive rate of microbial culture has not been satisfactory in the clinic due to the inherent shortcomings of culture. Second, the use rate of antibiotics before PICU admission was 65.8%. Third, the microorganisms that cause sepsis may be viruses or others, which can not be identified by ordinary culture. Finally, sepsis is a highly heterogeneous clinical diagnosis, and its manifestations are complex and changeable. Noninfectious etiologies, such as Kawasaki shock syndrome, hemophagocytic syndrome and macrophage activation syndrome, which may be misdiagnosed as sepsis, thus reducing the positive rate of culture. However, we found that there was no significant difference in comorbidities between the two groups.

In the past decade, many studies have explored the impact of culture results on the prognosis of adult patients with sepsis. Kim et al. (7) indicated that 90-day mortality and in-hospital mortality between the CNSS group and CPSS were not significantly different in adult patients. One meta-analysis study, including seven studies and enrolling 22,655 patients, also showed that the culture results were not associated with mortality in adult sepsis or septic shock patients (8). However, Gupta et al. (6) found that in-hospital mortality was significantly higher in adult patients with culture-negative severe sepsis than in those with culture-positive severe sepsis by using the Nationwide Inpatient Sample (NIS) database of the United States from 2000 to 2010. Different studies have shown inconsistent conclusions on the association between the culture results and mortality in sepsis patient. Of course, these differences may be due to revisions of the definition and diagnostic criteria of sepsis over time and differences in medical treatment level and the constituent ratio of patients with underlying diseases in different studies.

In the field of children's sepsis, there have been few studies on the impact of culture results on death. One study found that children with severe sepsis/septic shock with positive bacterial blood cultures had higher mortality than those with negative bacterial blood cultures (9). This study indicated that in-hospital mortality in children with CNSS was not significantly different compared with children with CPSS. Notably, we derived a relatively high mortality rate (47.1%) in this study. This may be related to the fact that the patient's condition was severe when the patient was admitted to the PICU. They had a high rate of receiving mechanical ventilation (66.4%) and/or renal replacement therapy (31.9%), and they also had high scores on the PRISM-III, PIM-3, and pSOFA scales.

Patients with severe sepsis/septic shock usually accompanied by respiratory failure, acute kidney injury and other organ dysfunction and need to be hospitalized for a long time. The LOS of hospital and MV duration were both longer in the culture-positive septic group than in the culture-negative septic group, while the CRRT duration and the requirements of MV and CRRT were similar between the two groups (9). Gupta et al. (6) found that the use rates of MV (37.7% vs. 35.7%) and AKI requiring dialysis (5.3% vs. 6.1%) in adult patients with culture-negative severe sepsis and culture-positive severe sepsis were similar; however, there were significant differences between the two groups because of the large sample size included in the study. In this study, we found that the LOS of hospital of children with CPSS was significantly longer than that of children with CNSS. One possible explanation for this result is that culture-positive children usually require a longer duration of antibiotic therapy, especially in the settings of bacteremia, abscess and osteomyelitis. In this study, the requirements and duration of MV and CRRT were not significantly different between the two groups, which indicated that the intensity of organ functional support therapy was similar in the CNSS group and the CPSS group.

Few articles in the current research focus on the relationship between serum sodium and culture results. Our study found that the serum sodium in the CPSS group was significantly lower than that in the CNSS group, which may mean that patients in the CPSS group need more aggressive measures to maintain electrolyte balance.

PCT may reflect the severity of infection, guide antimicrobial treatment and reduce unnecessary antibiotic use in clinic (18–20). Some studies have indicated that sepsis patients with elevated PCT levels have a higher mortality rate than patients with decreased PCT levels (21–23). Bakhtiar et al. (24) found that PCT was significantly higher in culture-positive sepsis than in culture-negative sepsis. In our study, PCT in the CPSS group was significantly higher than that in the CNSS group. Several studies have indicated that PCT levels are significantly higher in adult patients with gram-negative sepsis than in adult patients with gram-positive sepsis (25–28). However, in this study, subgroup analysis showed that there was no significant difference in PCT levels between gram-positive and gram-negative septic shock in children.

CRP is also a nonspecific inflammatory marker. Phua et al. (16) showed that the CRP level in culture-positive patients with severe sepsis was not significantly higher than that in culture-negative patients. However, Kim et al. (8) found that CPSS patients had significantly higher CRP levels than CNSS patients. Consistent with the study of Kim et al. (8), our study found that CRP in children with CPSS was significantly higher than that in children with CNSS. After removing children with hematological malignancies, the CPSS group still had higher levels of PCT and CRP than the CNSS group. This study also demonstrated that children infected with gram-positive bacteria have higher CRP, which may help pediatrician make better decision on antibiotic selection.

In this study, no significant difference in clinical outcomes between the two groups, while hospital stays were indeed longer in the CPSS group. Our study hinted that significantly elevated PCT and CRP may be related to positive culture results and prolonged hospital stay in children with septic shock. The high mortality of sepsis/septic shock may be attributed to the heterogeneity, that is, the complexity and diversity of sepsis/septic shock from etiology, pathogenesis to clinical outcome. With the deepening understanding of sepsis heterogeneity, it is necessary to classify sepsis from its clinical phenotype. It may be of great significance to analyze the subtype characteristics, treatment needs and inflammatory response of pediatric septic shock from the aspects of bacterial culture results or infection sites. However, there are few researches on these aspects. Exploring the subgroup characteristics of children with septic shock may be able to standardize the definition of pediatric sepsis, find more targeted and effective treatment measures, and better predict the prognosis.

## Limitations

There are several limitations in this study. First, the findings were retrospectively derived from a single center and at risk of bias. Second, the size of the samples included in the study was relatively small. Third, the high rate of antibiotic use before PICU admission may have affected the culture results. Fourth, children with hematological malignancies accounted for 18.9% of all patients, which may have had a certain impact on blood test indicators. After excluding such children, we still found that there was no change in statistical trends. Sixth, in this study, a considerable number of children who admitted to the PICU had multiple organ dysfunctions. Therefore, the patients included in our study may be more serious than other studies. Finally, although the PICU medical staff were experienced in culture sample collection, it was difficult to guarantee whether each culture-negative or -positive specimen was strictly qualified because of the inherent defect of retrospective research. However, this may be the first clinical study concerning the relationship between the culture results and the clinical outcome in children with septic shock in the PICU.

## Conclusion

Approximately 47.9% of children with septic shock were culture-positive. Children with CPSS had higher PCT and CRP levels, but lower WBC count and serum sodium. And the length of hospital stay of children with CPSS was longer than that of children with CNSS. However, in-hospital mortality, the length of PICU stay, the requirements of MV and CRRT were not associated with positive or negative culture results in children with septic shock in the PICU.

## Data Availability

The raw data supporting the conclusions of this article will be made available by the authors, without undue reservation.
