# Supplementary material for: Comparing outcomes between culture-positive and culture-negative septic shock in a PICU: A retrospective cohort study
Source: Front Pediatr. 2022 Oct 13;10:1001565. doi: 10.3389/fped.2022.1001565 (PMC9608626; doi:10.3389/fped.2022.1001565)
Supplement: Supplementary file 1 [file Table1.docx]

**Supplementary Table 1: Demographics, clinical characteristics of children with septic shock according to the culture results exclusion of 45 hematologic malignancy**

| **Characteristics** | **Total(N=193)** | **CNSS(n=107)** | **CPSS(n=86)** | **P** |
| --- | --- | --- | --- | --- |
| Age(month) , M(IQR) | 24(7~96) | 24(8~96) | 24(6~98) | 0.557 |
| Gender(male), n(%) | 106(54.9%) | 59(52.3%) | 47(54.7%) | 0.946 |
| Comorbidity, n(%) |  |  |  |  |
| Tumor | 11(5.7%) | 6(5.6%) | 5(5.8%) | 0.951 |
| Immunodeficiency | 12(6.2%) | 5(4.7%) | 7(8.1%) | 0.322 |
| Rheumatism | 24(12.4%) | 16(15.0%) | 8(9.3%) | 0.237 |
| PRISM-III, M(IQR) | 10(5~17) | 10(5~16) | 11(6~17) | 0.591 |
| PIM-3, M(IQR) | 0.08(0.05~0.16) | 0.08(0.04~0.17) | 0.08(0.05~0.14) | 0.827 |
| pSOFA, M(IQR) | 9(6~14) | 10(6~14) | 9(6~14) | 0.838 |
| WBC(x109/L) , M(IQR) | 9.4(3.97~17.46) | 10.14(5.11~21.16) | 8.03(2.94~15.90) | 0.046 |
| Hb(g/L) , M(IQR) | 98(83~118) | 100(84~119) | 96(80~114) | 0.108 |
| PLT(x109/L) , M(IQR) | 154(69~289) | 174(69~345) | 143(67~242) | 0.061 |
| CRP(mg/L) , M(IQR) | 41(13~76) | 26(4~66) | 59(23~9) | 0.002 |
| PCT(ng/mL) , M(IQR) | 19.01(3.29~79.14) | 9.62(2.00~77.99) | 34.63(7.86~78.14) | 0.003 |
| INR, M(IQR) | 1.38(1.15~1.91) | 1.38(1.10~1.94) | 1.38(1.19~1.86) | 0.600 |
| Lactate( mmol/L) , M(IQR) | 2.2(1.0~4.4) | 2.0(1.0~4.4) | 2.0(1.0~4.2) | 0.769 |
| Glucose(mmol/L) , M(IQR) | 6.0(4.8~7.9) | 6.0(4.8~8.3) | 5.9(4.8~7.3) | 0.440 |
| K^+^（mmol/L）, M(IQR) | 3.7(3.3~4.4) | 3.7(3.3~4.5) | 3.7(3.2~4.2) | 0.465 |
| Na^+^（mmol/L）, M(IQR) | 135(130~139) | 137(131~140) | 132(128~137) | 0.003 |
| Bilirubin（μmol/L）, M(IQR) | 6.60(3.35~15.15) | 5.60(2.90~13.20) | 8.20(3.65~18.10) | 0.128 |
| ALT（U/L）, M(IQR) | 48.5(28.8~120.6) | 48.5(30.2~127.1) | 51.8(26.5~116.2) | 0.389 |
| Albumin（g/L）, M(IQR) | 26.2(22.9~32.3) | 27.2(23.2~32.8) | 25.2(22.5~31.5) | 0.158 |
| Creatinine(μmoI/L) , M(IQR) | 47.9(27.7~85.0) | 47.9(~29.0~100.0) | 47.3(25.3~80.9) | 0.423 |
| Urea(mmol/L) , M(IQR) | 6.48(4.11~10.76) | 6.13(4.13~11.69) | 6.74(4.02~9.78) | 0.961 |
| Antibiotic use before PICU, n(%) | 125(64.8%) | 69(64.5%) | 56(65.1%) | 0.927 |
| Use of vasoactive drugs within 24 hours, n(%) | 148(76.7%) | 81(75.7%) | 67(77.9%) | 0.719 |
| Need of CRRT, n(%) | 64(33.2%) | 33(30.8%) | 31(36.0%) | 0.445 |
| The length of CRRT(day) , M(IQR) | 2(1~4) | 2(1~4) | 3(2~5) | 0.104 |
| Need of MV, n(%) | 135(69.9%) | 70(65.4%) | 65(75.6%) | 0.126 |
| The length of MV(day) , M(IQR) | 5(1~9) | 3(1~8) | 5(2~11) | 0.033 |

CNSS, culture-negative septic shock; CPSS, culture-positive septic shock; CRP, C-reactive protein; CRRT, continuous renal replacement therapy; Hb,:hemoglobin; INR, international normalized ratio; IQR, interquartile range; K^+^, blood potassium; M, median; MV, mechanical ventilation; Na+, serum sodium; PLT, platelet; PCT:procalcitonin, pSOFA, pediatric sequential organ failure assessment; WBC, white blood cells.
