# Supplementary material for: Comparing outcomes between culture-positive and culture-negative septic shock in a PICU: A retrospective cohort study
Source: Front Pediatr. 2022 Oct 13;10:1001565. doi: 10.3389/fped.2022.1001565 (PMC9608626; doi:10.3389/fped.2022.1001565)
Supplement: Supplementary file 2 [file Table2.docx]

**Supplementary Table 2: Clinical outcomes according to the culture results after exclusion of 45 hematologic malignancy**

| **Characteristics** | **Total(n=193)** | **CNSS(n=107)** | **CPSS(n=86)** | **P** |
| --- | --- | --- | --- | --- |
| LOS of PICU (days) , M(IQR) | 5(2~11) | 4(1~9) | 7(3~13) | 0.004 |
| LOS of hospital (days) , M(IQR) | 13(3~23) | 10(2~19) | 15(6~29) | 0.007 |
| 14-day PICU-free days, M(IQR) | 0(0~7) | 0(0~9) | 0(0~5) | 0.297 |
| 28-day hospital-free days, M(IQR) | 0(0~12) | 0(0~12) | 0(0~7) | 0.041 |
| In-hospital mortality, n(%) | 82(42.5%) | 46(43.0%) | 36(41.9%) | 0.875 |

IIQR, interquartile range; LOS, length of stay; M, median; PICU, pediatric intensive care unit.
